# Fascin promotes migration and invasion and is a prognostic marker for oral squamous cell carcinoma

**DOI:** 10.18632/oncotarget.20360

**Published:** 2017-08-19

**Authors:** Priscila Campioni Rodrigues, Iris Sawazaki-Calone, Carine Ervolino de Oliveira, Carolina Carneiro Soares Macedo, Mauricio Rocha Dourado, Nilva K. Cervigne, Marcia Costa Miguel, Andreia Ferreira do Carmo, Daniel W. Lambert, Edgard Graner, Sabrina Daniela da Silva, Moulay A. Alaoui-Jamali, Adriana Franco Paes Leme, Tuula A. Salo, Ricardo D. Coletta

**Affiliations:** ^1^ Department of Oral Diagnosis, School of Dentistry, University of Campinas, Piracicaba, SP, Brazil; ^2^ Unit of Cancer Research and Translational Medicine, Faculty of Medicine and Medical Research Center Oulu, Oulu University Hospital, University of Oulu, Oulu, Finland; ^3^ Oral Pathology and Oral Medicine, Dentistry School, Western Paraná State University, Cascavel, PR, Brazil; ^4^ Department of Dentistry, Federal University of Rio Grande do Norte, Natal, RN, Brazil; ^5^ Integrated Biosciences, School of Clinical Dentistry and Sheffield Cancer Centre, University of Sheffield, Sheffield, United Kingdom; ^6^ Departments of Medicine, Oncology, Pharmacology and Therapeutics, Segal Cancer Centre and Lady Davis Institute for Medical Research, Sir Mortimer B. Davis-Jewish General Hospital, Montreal, Quebec, Canada; ^7^ Otolaryngology-Head and Neck Surgery, Faculty of Medicine, McGill University, Montreal, Quebec, Canada; ^8^ Brazilian Biosciences National Laboratory-CNPEM, Campinas, SP, Brazil; ^9^ Institute of Oral and Maxillofacial Disease, University of Helsinki, and HUSLAB, Department of Pathology, Helsinki University Hospital, Helsinki, Finland; ^10^ Current/Present address: Clinical Department, Faculty of Medicine of Jundiai, Jundiai, SP, Brazil

**Keywords:** fascin, plectin, oral squamous cell carcinoma, migration and invasion, prognosis

## Abstract

Oral squamous cell carcinoma (OSCC) prognosis is related to clinical stage and histological grade. However, this stratification needs to be refined. We conducted a comparative proteome study in microdissected samples from normal oral mucosa and OSCC to identify biomarkers for malignancy. Fascin and plectin were identified as differently expressed and both are implicated in several malignancies, but the clinical impacts of aberrant fascin and plectin expression in OSCCs remains largely unknown. Immunohistochemistry and real-time quantitative PCR were carried out in *ex vivo* OSCC samples and cell lines. A loss-of-function strategy using shRNA targeting fascin was employed to investigate *in vitro* and *in vivo* the fascin role on oral tumorigenesis. Transfections of microRNA mimics were performed to determine whether the fascin overexpression is regulated by miR-138 and miR-145. We found that fascin and plectin are frequently upregulated in OSCC samples and cell lines, but only fascin overexpression is an independent unfavorable prognostic indicator of disease-specific survival. In combination with advanced T stage, high fascin level is also an independent factor of disease-free survival. Knockdown of fascin in OSCC cells promoted cell adhesion and inhibited migration, invasion and EMT, and forced expression of miR-138 in OSCC cells significantly decreased the expression of fascin. In addition, fascin downregulation leads to reduced filopodia formation and decrease on paxillin expression. The subcutaneous xenograft model showed that tumors formed in the presence of low levels of fascin were significantly smaller compared to those formed with high fascin levels. Collectively, our findings suggest that fascin expression correlates with disease progression and may serve as a prognostic marker and therapeutic target for patients with OSCC.

## INTRODUCTION

Oral squamous cell carcinoma (OSCC) is the eleventh most commonly diagnosed cancer worldwide, accounting for 300,000 new cases and 145,000 deaths per year [1]. The most relevant OSCC prognostic factor is clinical staging of the disease based on TNM classification (tumor size, lymph node spread and distant metastasis), however, the behavior of some OSCCs are uncertain [2, 3]. Besides, histological scoring systems are constantly revised, and in numerous instances new scoring systems have shown an important role in prognostication of patients with OSCC [4, 5]. Surgery remains the mainstay of curative treatment, while the response to chemo and radiotherapy is limited. Owing to late diagnosis and frequent development of locoregional recurrences and second primary tumors, mortality rates are 50% over 5 years and have remained unchanged over recent decades [6]. At advanced stage, OSCC has an extremely poor prognosis. Therefore, the identification of novel biomarkers for early detection, post-therapeutic monitoring and to facilitate development of novel therapeutic approaches is of great importance.

Fascin and plectin are cytoskeleton-binding proteins associated with cell motility in both normal and neoplastic conditions [7]. Fascin bundles actin filaments within dynamic cellular structures such as microspikes, stress fibers and membrane ruffles [8]. The expression of fascin is low or absent in adult epithelial cells, but its overexpression in tumors is associated with poor prognosis [9]. Functional studies revealed that fascin has the ability to promote migration and invasion of carcinoma cells *in vitro* [10], and its expression is associated with increased invasive and metastatic potential in mouse xenograft tumor models [11]. In OSCCs, fascin expression level was associated with aggressiveness [12, 13] and an *in vitro* study showed that fascin regulates epithelial-mesenchymal transition (EMT) and invasion of OSCC cells [14]. Plectin is important to maintain intracellular architectures and the normal cellular morphology after binding to cytoskeletal proteins (reviewed in [15]). Plectin also mediates the polymerization of fibronectin fibrils while fibrillar adhesions occur [16], and participates in regulation of cell migration and invasion through activation of ERK1/2 kinase [7, 17]. Although few studies have demonstrated overexpression in cancer cells, the participation and the mechanisms of action and regulation of plectin in cancer remain elusive. The study by Katada and collaborators [17] showed that plectin levels are correlated with proliferation, migration, invasion and poor prognosis in head and neck squamous cell carcinomas. With the exception of this study, little has been uncovered regarding the biological mechanisms related to plectin in oral cancer. Although the emerging literature suggests the clinical importance of fascin and plectin on human cancers as potential prognostic markers or therapeutic targets, there are still very little molecular details defining the mechanisms of action of those proteins in the control of oral tumorigenesis.

Laser-capture microdissection associated with mass spectrometry-based proteomics analysis (LC-MS/MS) conducted by our group revealed that fascin and plectin are overexpressed in OSCC tissues in comparison with oral healthy mucosas [18]. In the present study, we examined the expression levels of fascin and plectin in OSCC clinical samples and cell lines to determine the prognostic impact of those proteins for OSCC patients. Moreover, we assessed whether fascin knockdown influences OSCC cell proliferation, adhesion, migration, invasion, EMT and filopodia formation *in vitro*. The influence of modulating fascin expression *in vivo* was examined using OSCC tumor formation and cervical lymph node metastasis models. Furthermore, to understand the molecular mechanism through which fascin is overexpressed, the regulation of fascin expression by miR-138 and miR-145 was investigated in OSCC cell lines and clinical specimens.

## RESULTS

### Fascin and plectin are overexpressed in OSCC tissues and cell lines

**Figure 1:**
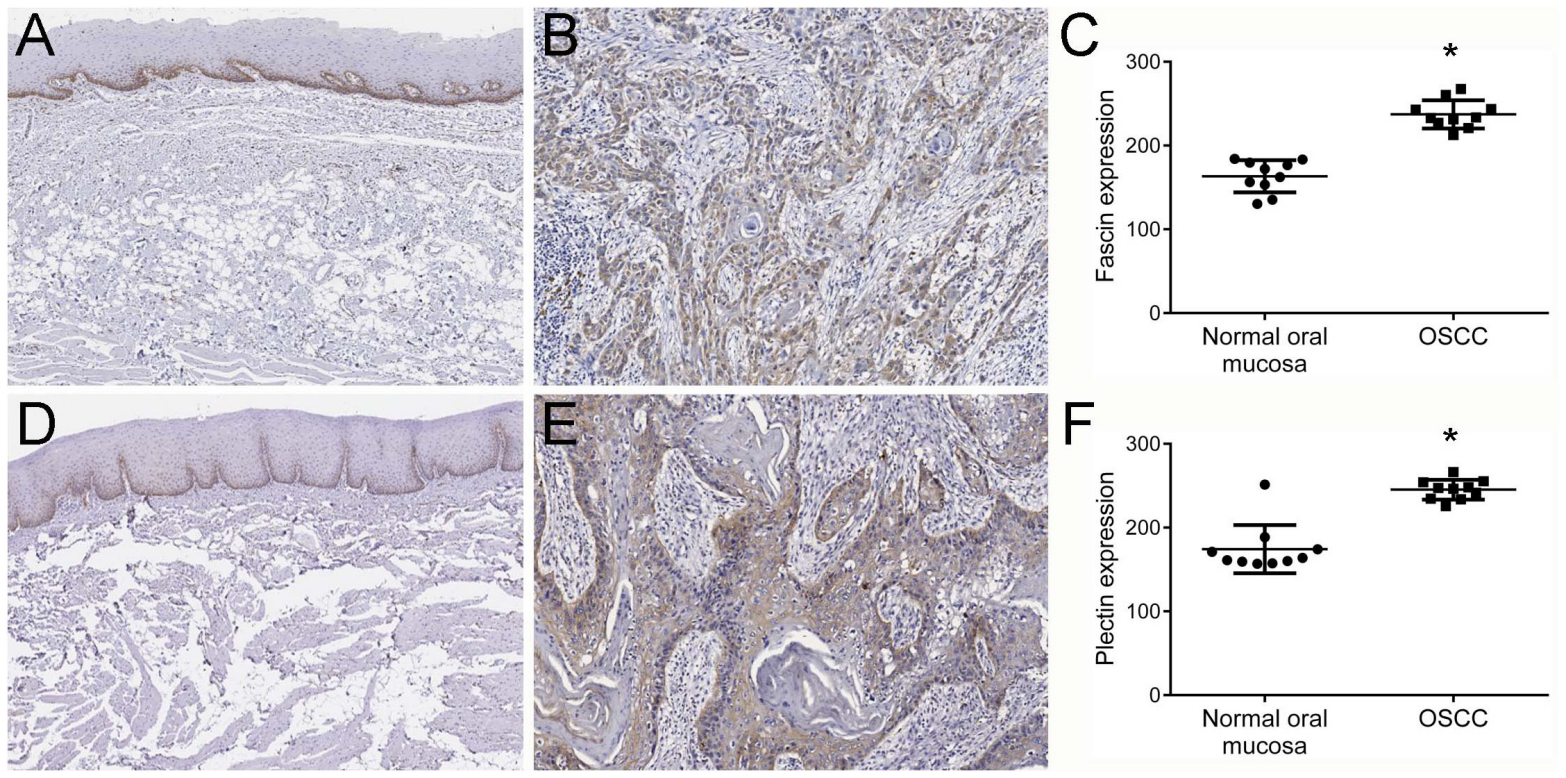
Higher expression levels of fascin and plectin in OSCCs Representative immunohistochemical expression patterns of fascin and plectin in normal oral mucosa and OSCC specimens are shown. Both fascin **(A)** and plectin **(D)** expression were limited to the cytoplasm of the basal and suprabasal layers of the normal oral tissue. OSCC tumor cells showed variable distribution and intensity of fascin **(B)** and plectin **(E)**. Quantification of the positive expression of both fascin **(C)** and plectin **(F)** in the ten original pairs of samples used in the LC–MS/MS revealed a significantly higher expression in OSCC cells compared with normal oral mucosa cells *p<0.0001.

Firstly, fascin and plectin expression levels were validated in the same cohort used in the previously reported LC-MS/MS analyses [18] to confirm that both proteins are overexpressed in OSCCs compared to normal tissues. Immunostaining for both fascin and plectin showed a cytoplasmic pattern (Figure 1). Normal epithelium revealed weak or partly moderate staining restricted to the lower layers for both fascin (Figure 1A) and plectin (Figure 1D), whereas tumor cells showed variable distribution and intensity of fascin (Figure 1B) and plectin (Figure 1E). Immunopositivity for fascin was also found in the endothelial cells, and plectin immunoreactivity was detected in some inflammatory cells of the stroma. Analysis of intensity of staining showed that both fascin (p<0.0001, Figure 1C) and plectin (p<0.0001, Figure 1F) were significantly more abundantly expressed in OSCCs than in control mucosas.

**Figure 2:**
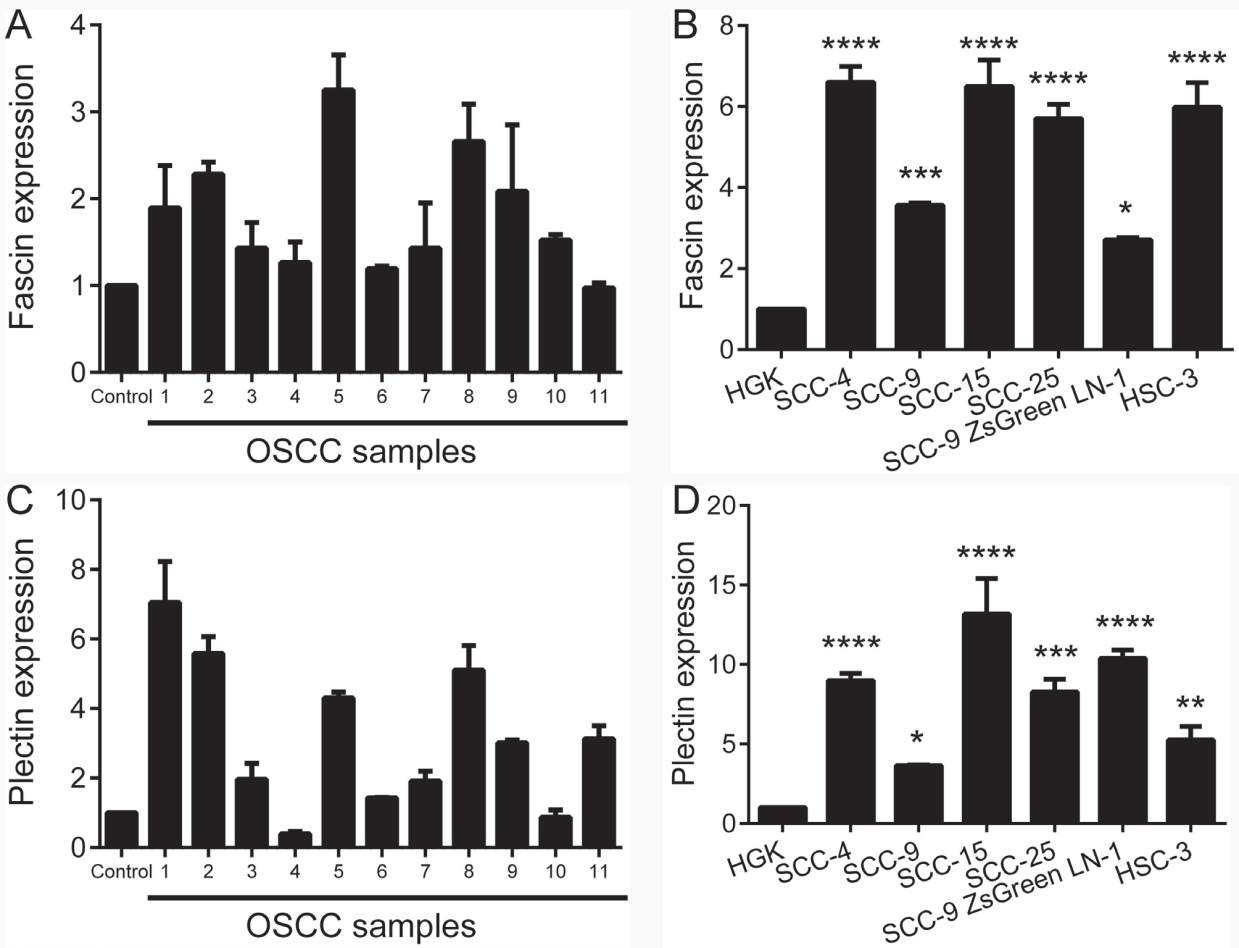
Fascin and plectin are overexpressed in OSCCs and OSCC-derived cell lines Total RNA from fresh samples and cell lines were converted in cDNA and subjected to qPCR. **(A)** In the fresh samples, the comparison was based in a pool of 11 normal oral tissues, whereas the spontaneously immortalized, but not transformed epithelial cell line HGK was used as reference for the comparison with OSCC-derived cell lines. The amounts of fascin (p<0.01; A) and plectin (p<0.05; C) mRNA were significantly higher in OSCC specimens than in the normal oral mucosa. The mRNA levels of fascin **(B)** and plectin **(D)** were also significantly higher in OSCC cell lines compared to HGK cells. *p<0.05, **p<0.01, ***p<0.001, ****p<0.0001.

The expression of fascin and plectin was analyzed by qPCR in fresh samples from normal oral mucosa and OSCC. In this experiment, we pooled 11 normal oral mucosa and used these as a reference. The variation on fascin and plectin expression levels was small among control samples. A significantly higher level of fascin mRNA was observed in the group of fresh tumors in comparison to reference pool (p<0.01, Figure 2A) and one sample showed fascin level similar to normal control. The mRNA levels of plectin were also significantly higher in the group of OSCC samples (p<0.05, Figure 2C), but 2 tumor samples showed lower plectin levels than the normal mucosa reference pool. The levels of fascin mRNA were significantly higher in the OSCC cell lines SCC-4 (p<0.0001), SCC-9 (p<0.001), SCC-15 (p<0.0001), SCC-25 (p<0.0001), SCC-9 ZsGreen LN-1 (p<0.01) and HSC-3 (p<0.001) compared with the spontaneously immortalized, but not transformed epithelial cell line HGK (Figure 2B). Similarly, plectin was significantly more abundantly expressed in the SCC-4 (p<0.0001), SCC-9 (p<0.05), SCC-15 (p<0.0001), SCC-25 (p<0.0001), SCC-9 ZsGreen LN-1 (p<0.001) and HSC-3 (p<0.01) than in the HGK (Figure 2D).

**Figure 3:**
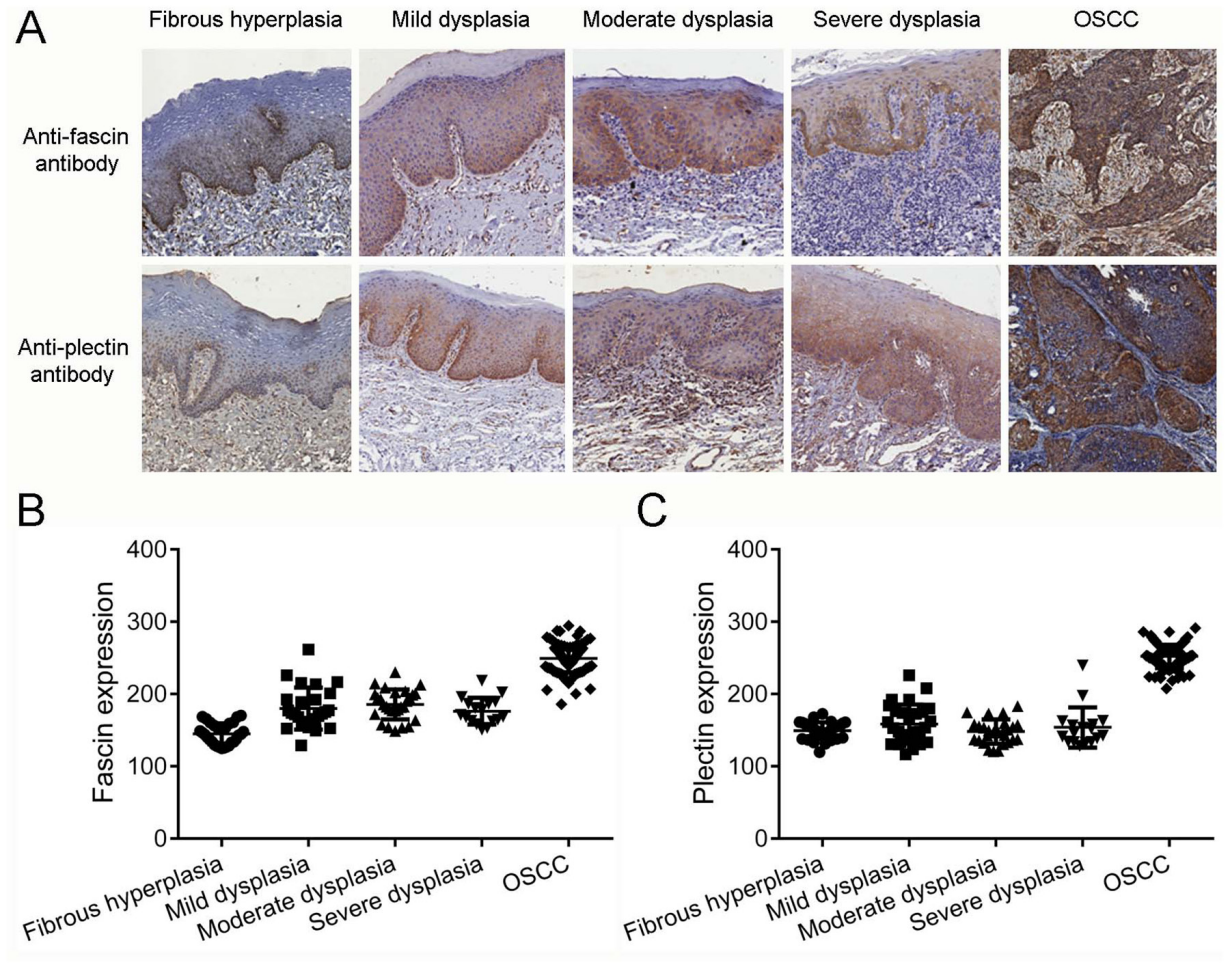
Fascin but not plectin is overexpressed in oral dysplasias **(A)** Immunodetection of fascin and plectin in representative samples of oral fibrous hyperplasia, characterizing normal oral epithelium, oral dysplasia and OSCCs. The expressions of fascin and plectin were not restricted to the lower layers of the epithelium in the oral dysplasias. **(B)** As expected, the levels of fascin were significantly higher in OSCCs than in fibrous hyperplasia (p<0.0001). Fascin was also significantly upregulated in the OSCCs when compared with dysplasia samples, independently of the grade (p<0.001). In addition, fascin protein levels were significantly higher in dysplasias than in nontumor tissues (p<0.001). **(C)** The levels of plectin were significantly higher in OSCCs than in fibrous hyperplasias and dysplasias (p<0.0001), but no differences between fibrous hyperplasia and dysplasias were observed.

The fascin protein levels were significantly higher in OSCCs than in fibrous hyperplasias (p<0.0001) and in dysplasias, independent of the grade (p<0.001; Figure 3A and 3B). Interesting, the expression of fascin in oral dysplasias was not limited to the lower layers of the epithelium (Figure 3A). Fascin was also significantly upregulated in the dysplasia samples when compared with fibrous hyperplasias (p<0.001; Figure 3B). The levels of plectin were significantly higher in OSCCs than in fibrous hyperplasias and dysplasias (p<0.0001; Figure 3C and 3D). However, no differences between fibrous hyperplasia and dysplasias were observed (Figure 3D). Thus, these data demonstrate that both fascin and plectin are frequently upregulated in OSCC tissues and cell lines.

### Expression of fascin is associated with survival of patients with OSCC

To evaluate the correlation between protein levels of fascin and plectin and clinicopathological characteristics, the patients of OSCC cohort 1 were divided into low and high expression subgroups with the median value as the cutoff. As shown in Table 1, the upregulation of fascin in OSCC tissues was not correlated with the clinicopathological features of tumors, while a significant correlation between plectin levels and involvement of the surgical margin was found (p=0.04).

**Table 1:** Spearman correlation between immunohistochemical expression of fascin and plectin and clinicopathological variables

Variables	Fascin	Plectin
	Correlation coefficient / p value	Correlation coefficient / p value
Age	0.082 / 0.39	0.171 / 0.07
Gender	-0.011 / 0.90	0.143 / 0.13
Smoking habit	-0.080 / 0.45	0.005 / 0.96
Drinking habit	0.088 / 0.43	0.001 / 0.99
Tumor site	0.083 / 0.38	-0.059 / 0.53
T stage	0.051 / 0.60	-0.113 / 0.23
N stage	0.135 / 0.16	-0.063 / 0.51
Treatment	0.092 / 0.33	-0.021 / 0.82
Histopathological grade	0.158 / 0.09	0.138 / 0.15
Margin Status	-0.101 / 0.29	0.194 / 0.04
Local recurrence	0.155 / 0.10	0.003 / 0.69
Regional recurrence	0.018 / 0.85	-0.031 / 0.74
Distant recurrence	-0.003 / 0.96	0.134 / 0.16
Second primary	-0.122 / 0.20	0.016 / 0.86

**Figure 4:**
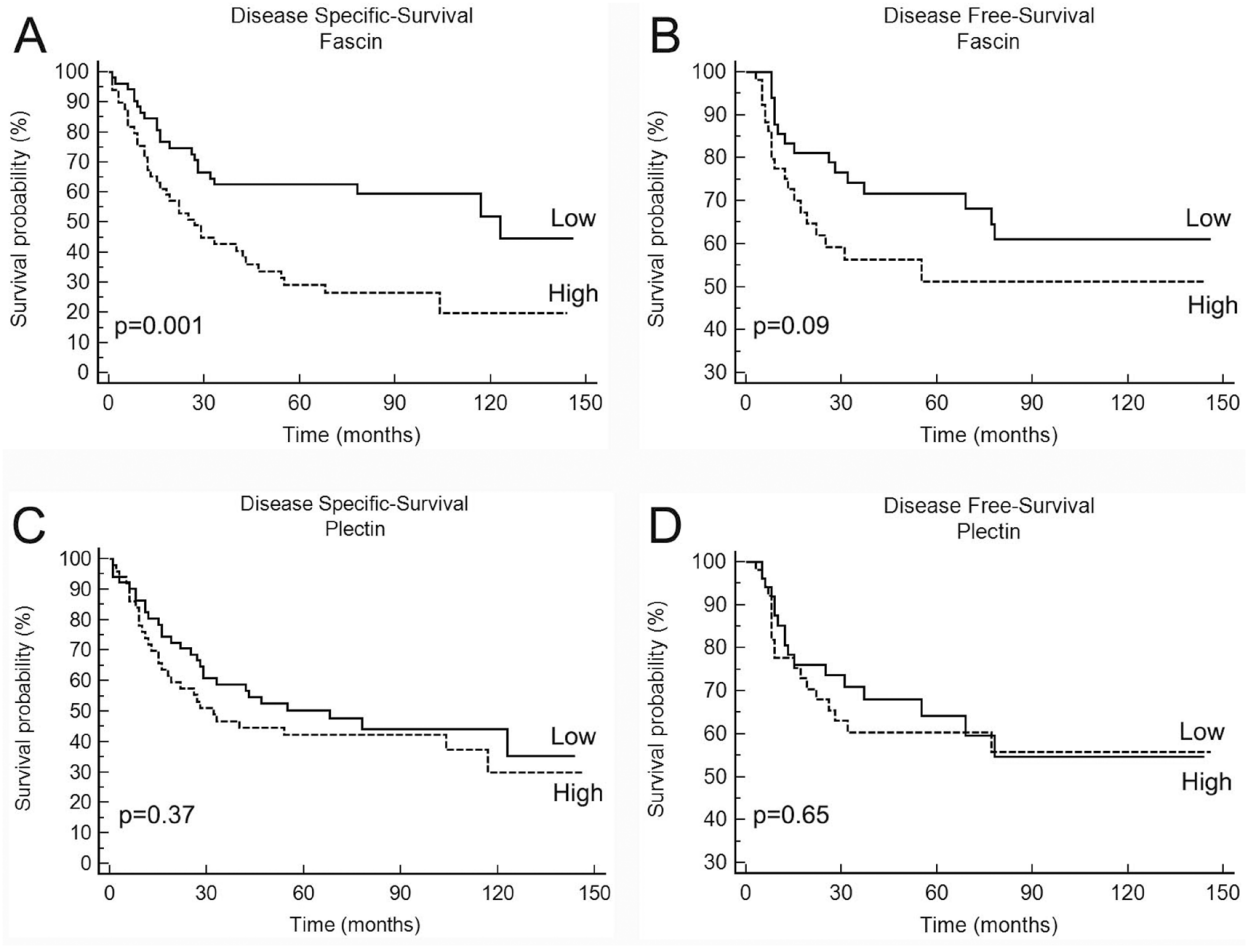
Kaplan-Meier cumulative curves for disease-specific and disease-free survivals of patients with OSCC according to expression of fascin **(A** and **B)** and plectin **(C** and **D)**. Patients with high fascin expression corresponded to those with a significantly poorer outcome in disease-specific survival.

We next assessed the association between expression of fascin and plectin and clinical prognosis of OSCC patients (cohort 1). The outcomes were categorized as disease-specific survival, time from treatment initiation until death due to cancer or last known date alive, and disease-free survival, time from treatment initiation until diagnosis of the first recurrence (local, regional or distant) or last follow-up information for those without recurrence. Patients with higher fascin expression had significantly poorer disease-specific survival (p<0.001) rate than those with lower fascin expression (Figure 4A). High fascin immunoreactivity showed a tendency towards association with shortened disease-free survival (Figure 4B). Recurrence was diagnosed in 48.8% of patients with strong positivity for fascin after 5-year follow up compared with 28.4% for those with low fascin expression (p=0.09; Figure 4B). For plectin, survival analyses based on univariate log-rank test revealed no significant association with both disease-specific survival (Figure 4C) and disease-free survival (Figure 4D). The univariate analysis for disease-specific survival and disease-free survival of the OSCC cohort is provided as Supplementary Materials (Supplementary Figures 1 and 2).

Multivariate Cox regression analyses confirmed that fascin expression level, together with patient age, size of the primary tumor (T stage) and regional metastasis at diagnosis (N stage), was an independent risk factor for disease-specific survival in this cohort (Table 2). A HR of 2.86 (95% CI: 1.34-6.12, p=0.007) was found for high expression of fascin in relation to low expression.

**Table 2:** Cox multivariate analysis for the risk of death

Parameter	Disease-specific survival		Disease-free survival	
	HR (95% CI)	p value	HR (95% CI)	p value
Age				
≤ 56	Reference			
> 56	2.43 (1.24±4.76)	0.01		
T stage				
T1/T2	Reference		Reference	
T3/T4	2.31 (1.41±5.89)	0.003	2.05 (1.46-7.91)	0.04
N stage				
N0	Reference			
N+	2.11 (1.20±4.30)	0.01		
Fascin levels				
Low	Reference			
High	2.86 (1.34±6.12)	0.007		

In order to strength the prognostic information of these independent factors, we combined fascin expression levels with age, T stage and N stage and performed univariate and multivariate survival analysis. There were significant associations of combinations of fascin levels and T stage in both DSS and DFS and of fascin levels and N stage in DSS (Table 3). In all combinations, the survival curves suggested a largely improved discriminatory ability. Interestingly, DFS was not significantly associated with fascin level alone, but when combined with T stage, a significant association was observed (HR: 4.17, 95% CI: 1.39-12.43, p=0.01), revealing an independent prognostic discrimination.

**Table 3:** Cox regression analysis for disease specific and disease-free survival for combination of fascin levels and age, T stage and N stage in the 113 oral squamous cell carcinoma patients

Parameters	Disease specific survival		Disease-free survival	
	Hazard ratio (95% CI) / p value		Hazard ratio (95% CI) / p value	
	Univariate	Multivariate	Univariate	Multivariate
Fascin and Age				
Low fascin and < 56	Reference		Reference	
High fascin or ≥ 56	1.88 (1.09-3.25) / 0.04		1.58 (0.78-3.20) / 0.24	
Fascin and T stage				
Low fascin and T1/T2	Reference	Reference	Reference	Reference
High fascin or T3/T4	3.45 (2.06-5.77) / <0.0001	4.82 (2.10-11.06) / 0.0002	2.07 (1.08-3.99) / 0.03	4.17 (1.39-12.43)/0.01
Fascin and N stage				
Low fascin and N0	Reference	Reference	Reference	
High fascin or N+	2.90 (1.69-4.98) / 0.002	4.89 (1.87-12.79) / 0.001	1.49 (0.75-2.94) / 0.27	

To confirm our findings, the expression levels of fascin were investigated in an independent set of OSCCs (cohort 2; details provided in materials and methods section). In agreement, univariate and multivariate survival analysis indicated that high levels of fascin were significantly associated with poor prognosis (p<0.05; Supplementary Figure 3). Collectively, these findings suggest that the expression level of fascin could be used as an independent factor to predict poor prognosis of OSCCs.

### Fascin knockdown suppresses the migratory and invasive potential of OSCC cells

**Figure 5:**
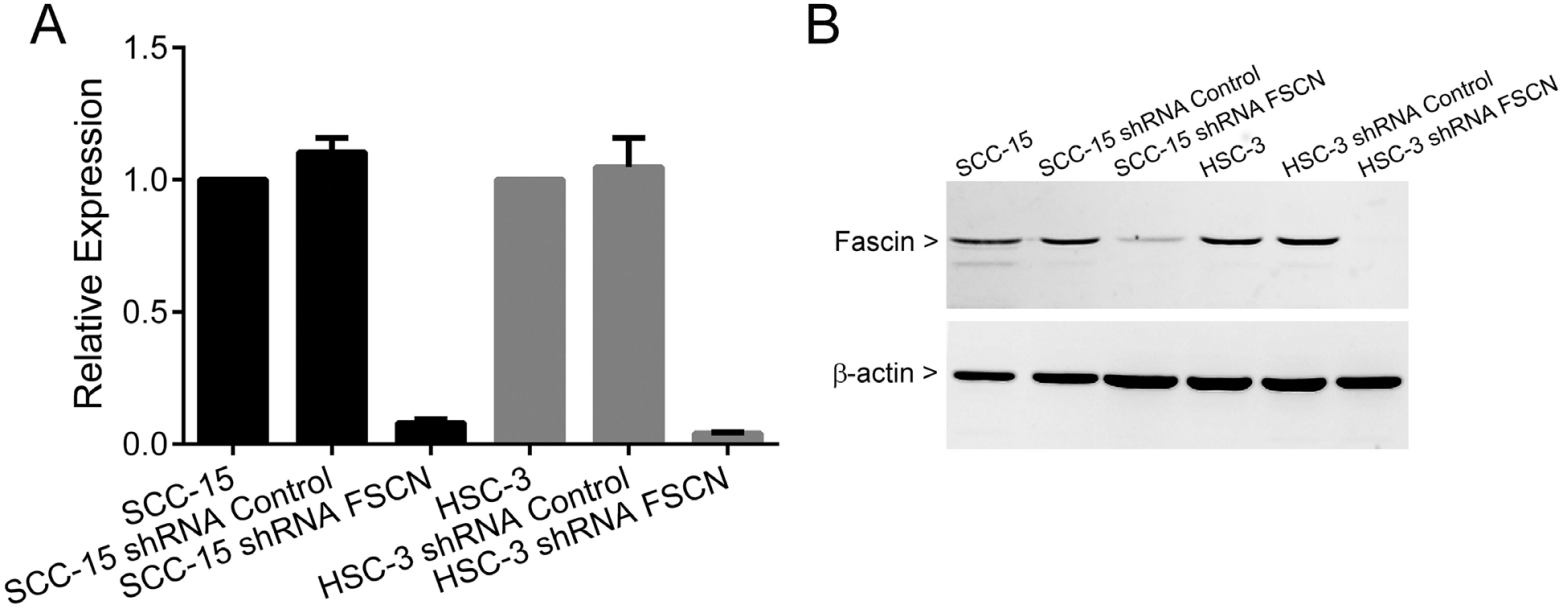
Fascin knockdown efficiency in SCC-15 and HSC-3 cells. Cells were transduced with lentivirus expressing shRNA sequences against fascin (shRNA FSCN cells) and control (shRNA Control cells) as outlined in the methods shRNA FSCN cells showed a marked reduction in both mRNA and protein levels when compared with parental cell and shRNA Control cells.

To examine the role of fascin in OSCC progression, fascin knockdown was achieved in the aggressive and highly invasive cell line HSC-3 and in the less invasive SCC-15, by lentivirus-mediated shRNA expression. Both cell lines have high levels of fascin expression. As expected, cells transduced with lentivirus carrying a specific sequence targeted to the fascin transcript demonstrated a significant reduction in both fascin mRNA and protein levels in comparison with parental cells or cells transduced with the control, non-targeting sequence (Figure 5).

**Figure 6:**
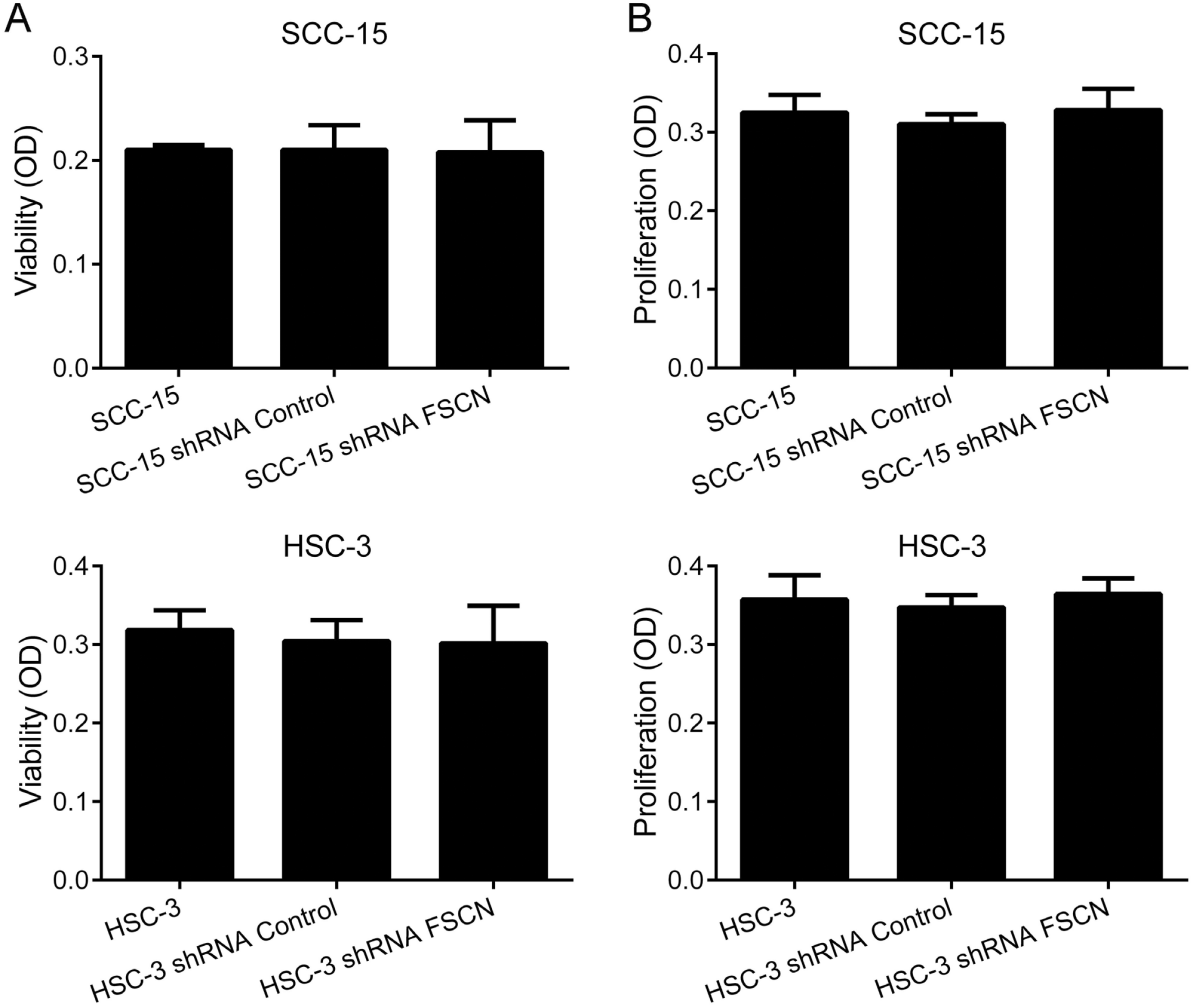
Downregulation of fascin does not affect viability or proliferation of SCC-15 and HSC-3 cells Cells were subjected to MTS-cell viability **(A** and **B)** and bromodeoxyuridine (BrdU)-labeling cell proliferation **(C** and **D)** assays.

**Figure 7:**
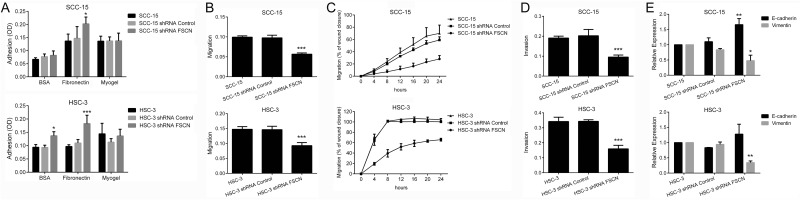
Overexpression of fascin is associated with adhesion, migration, invasion and acquisition of EMT properties **(A)** Downregulation of fascin significantly induced the adhesive properties of SCC-15 and HSC-3 cells to fibronectin, and of HSC-3 cells on uncoated surfaces. **(B)** Migration of SCC-15 and HSC-3 cells were significantly decreased by fascin-specific shRNA, as revealed by transwell migration assay. **(C)** Migration analysis based on scratch wound migration assay showed that fascin-silenced cells (SCC-15 shRNA FSCN and HSC-3 shRNA FSCN) closed the scratch wound significantly more slowly than parental cells (SCC-15 and HSC-3) and shRNA Control cells. **(D)** Invasion of SCC-15 and HSC-3 cells was significantly inhibited after fascin knockdown. **(E)** Downregulation of fascin induced significantly the expression of E-cadherin while reducing vimentin expression in SCC-15 cells. For HSC-3 cells, reduction of vimentin expression reached significant levels, while that the induction of E-cadherin did not. *p<0.05, **p<0.01, ***p<0.0001.

**Figure 8:**
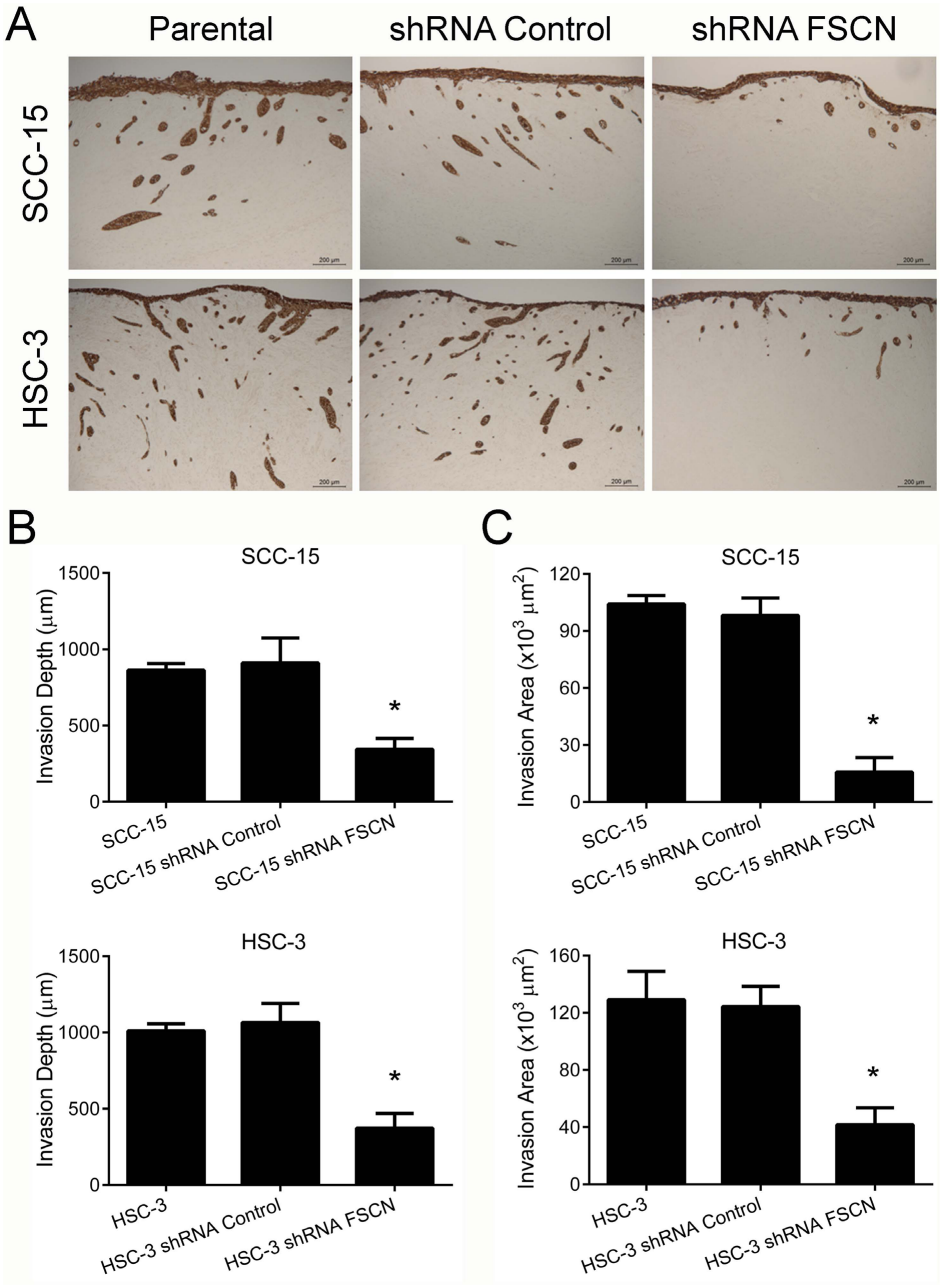
Fascin downregulation inhibits the invasion of SCC-15 and HSC-3 cells in the myoma organotypic invasion model **(A)** The knockdown of fascin markedly reduced the invasion properties of SCC-15 and HSC-3 cells in the myoma organotypic model when compared with control cells. The invasion depth **(B)** and the invasion area **(C)** were significantly reduced for HSC-3 shRNA FSCN cells. *p<0.0001.

Knockdown of fascin did not impair the viability or proliferation potential of the cells (Figure 6), but resulted in a significant increase in cell adhesion on surfaces coated with fibronectin for both SCC-15 (p<0.05) and HSC-3 cells (p<0.0001; Figure 7A). Transwell migration and invasion assays revealed that downregulation of fascin significantly inhibited the migratory (p<0.0001; Figure 7B) and invasive (p<0.0001; Figure 7D) ability of SCC-15 and HSC-3 cells. To further characterize the effects of fascin on migration and invasion, we carried out a scratch wound migration assay and a myoma organotypic invasion assay, respectively. As shown in Figure 7C, fascin-silenced cells (both SCC-15 and HSC-3) closed the scratch wound significantly more slowly than control cells (p<0.0001). Figure 8A illustrates the assessment of the invasive potential of the SCC-15 and HSC-3 cells in the myoma organotypic invasion assay. Invasion depth (Figure 8B) and invasion area (Figure 8C) were significantly reduced in both SCC-15 and HSC-3 expressing shRNA targeting FSCN compared with parental and control shRNA cells grown in the myoma discs (p<0.0001). To determine whether fascin influences EMT, we examined the expression levels of the epithelial marker E-cadherin and of the mesenchymal marker vimentin in the fascin-silenced cells. As indicated in Figure 7E, SCC-15 expressing FSCN-targeting shRNA had high mRNA levels of E-cadherin and reduced expression of vimentin compared to shRNA-control expressing cells, while HSC-3 cells expressing FSCN-targeting shRNA had reduced expression of vimentin, but had no significant increase on E-cadherin levels compared to shRNA-control expressing cells. Taken together, these results suggest that fascin is important for the migratory and invasive properties of OSCC cells.

### Xenograft tumors from the HSC-3 shRNA FSCN cells in nude mice

**Figure 9:**
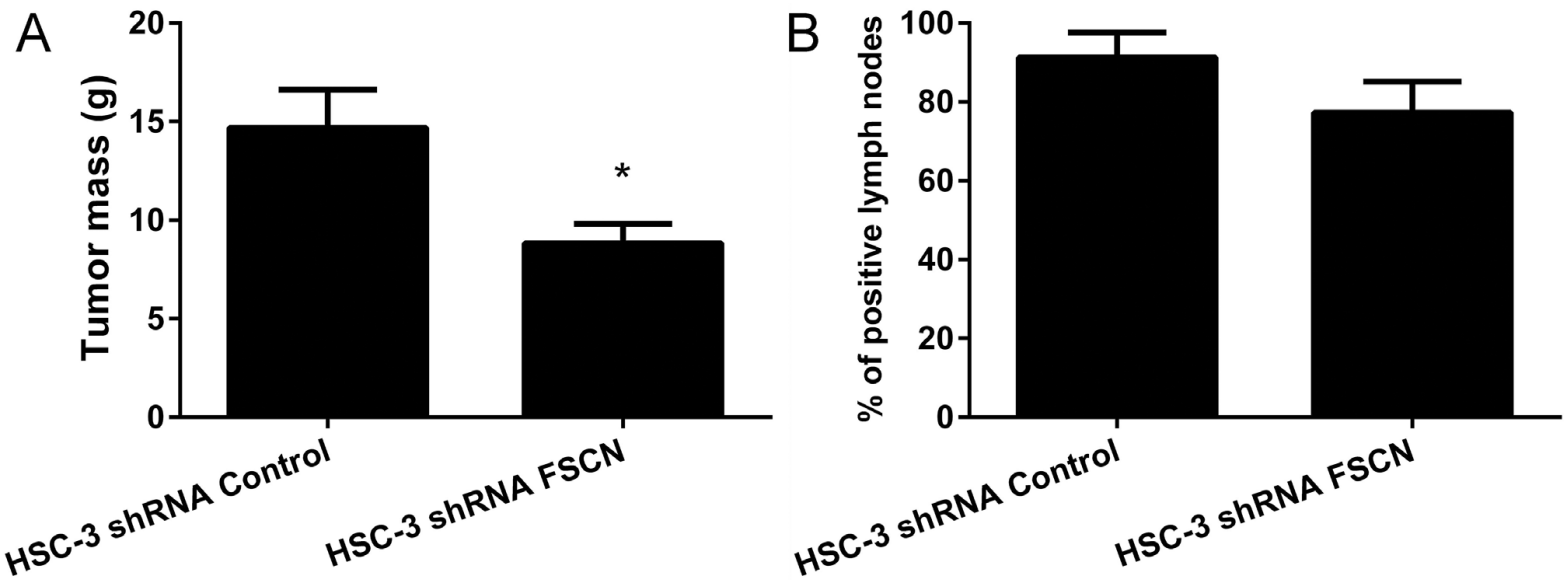
The mass of the tumors and the frequency of cervical lymph node metastasis in xenograft tumors formed by HSC-3 shRNA Control and HSC-3 shRNA FSCN cells **(A)** In the subcutaneous tumor model, HSC-3 shRNA FSCN tumors were significantly smaller than HSC-3 shRNA Control tumors. **(B)** In the orthotopic tongue model, no differences in the frequency of cervical lymph node metastasis were observed. *p<0.01.

Next, we assessed the potential action of fascin in tumorigenicity using HSC-3 xenograft tumors formed in the flank of BALB/c nude mice. The volume of the HSC-3 shRNA FSCN xenograft tumors was slightly decreased compared with tumors formed by the HSC-3 shRNA Control cells (p<0.01, Figure 9A). No macroscopic or microscopic differences were observed, but the immunohistochemical analysis confirmed fascin knockdown in the HSC-3 shRNA FSCN xenograft tumor cells in contrast with high expression levels in the HSC-3 shRNA Control tumors (Supplementary Figure 4). It is worth noting that some fascin immunostaining was detected on basal cells of tumor nests in HSC-3 shRNA FSCN xenograft tumors. Three weeks after the implantation of the cells into the tongue of BALB/c nude mice, all animals exhibited tumors characterized by a firm mass in the middle part of the tongue. Visible cervical lymph nodes were carefully removed and investigated for the presence of metastasis using immunohistochemistry for pan-cytokeratins. The frequency of lymph node metastases was lower in HSC-3 shRNA FSCN orthotopic tumors compared with HSC-3 shRNA Control tumors, but did not reach a statistically significant level (p=0.18, Figure 9B). As expected, the tongue tumors formed by HSC-3 shRNA FSCN cells showed no immunoexpression of fascin (Supplementary Figure 4).

### Downregulation of fascin ablates filopodia formation and reduces expression and phosphorylation of paxillin

**Figure 10:**
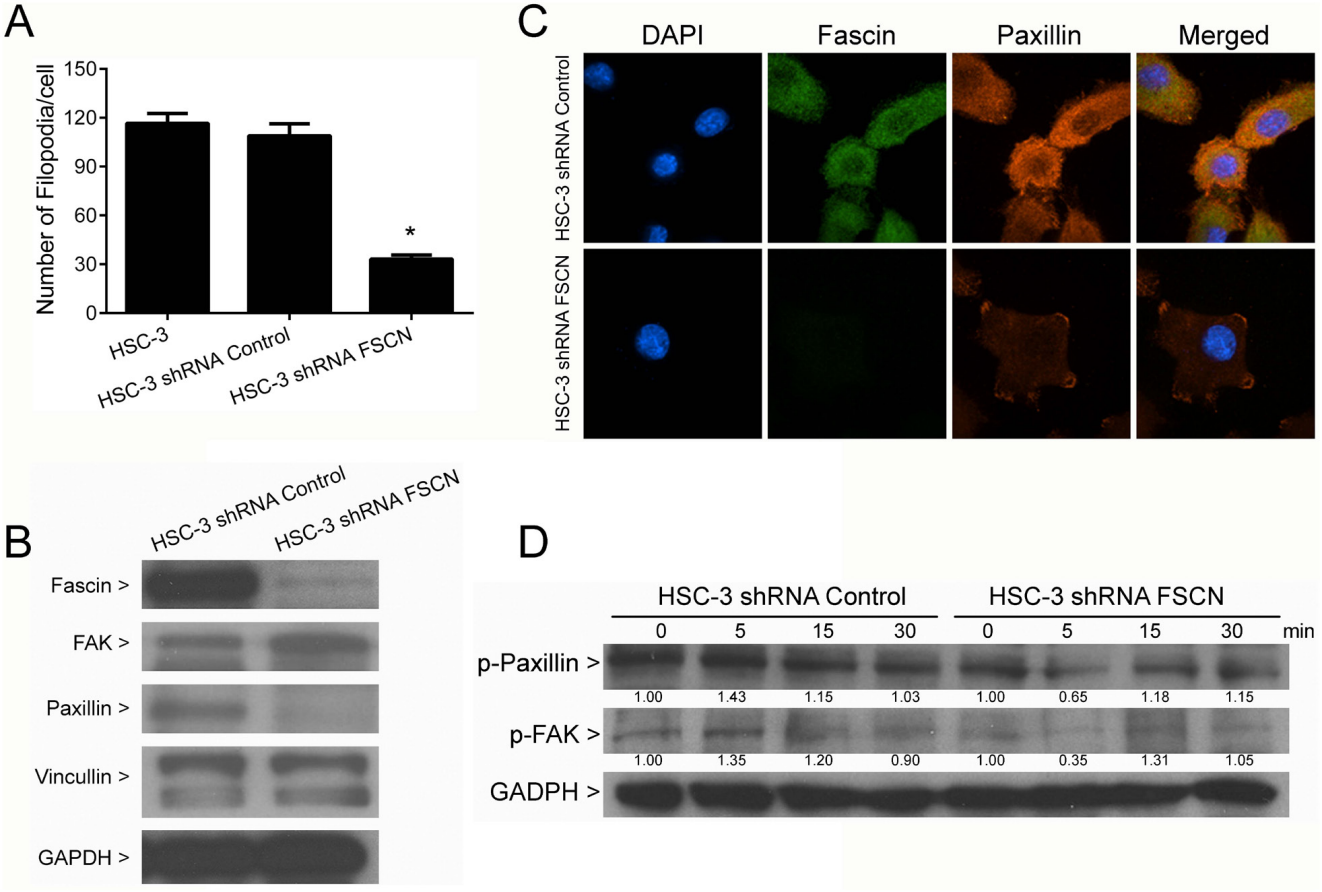
Inhibition of filopodia formation by fascin knockdown **(A)** Actin filaments were labeled with rodhamine phalloidin, and the number of filopodia structures was estimated with the aid of the Image J software. Fascin knockdown significantly reduced the number of filopodia (p<0.0001). **(B)** Western blot analysis of filopodia-related proteins. Fascin-silenced cells demonstrated reduced expression paxillin in comparison with control cells. No significant effects were observed in FAK and viculin. **(C)** Confocal analysis revealed the colocalization of fascin and paxillin, and confirmed the decreased expression of paxillin in fascin-silenced cells. **(D)** HSC-3 shRNA Control and HCS-3 shRNA FSCN were cultured in presence of 50 ng/ml of EGF for 5, 15 and 30 min, and assessed by western blot with antibodies against phospho-fascin, phospho-paxillin and phospho-FAK. Values above bands represent the densitometric analysis. Reduction in phosphorylation of paxillin and FAK occurs upon fascin-silencing in HSC-3 cells. *p<0.0001.

Since fascin is associated with the initiation and formation of filopodia structures, we considered whether fascin-silencing in HSC-3 cells may affect filopodia formation. As expected, fascin-depleted cells demonstrated significantly lower number of filopodia in comparison with control clones (p<0.0001, Figure 10A and Supplementary Figure 5). Subsequently we assessed whether fascin silencing could interfere in the expression of other proteins related to filopodia protrusions, including paxillin, vinculin and FAK. Fascin depletion was accompanied by a marked downregulation of paxillin, whereas no significant effects on vinculin and FAK expression could be observed (Figure 10B). Accordingly, fascin co-localized with paxillin in HSC-3 shRNA Control cells and both fascin and paxillin were downregulated in HSC-3 shRNA FSCN cells (Figure 10C). The fascin silenced cells stimulated with 50 ng/ml of EGF showed slight attenuation on paxillin and FAK phosphorylations in comparison with control (Figure 10D). These results suggest that fascin may have an important role in filopodia development.

### Fascin expression is inversely correlated with miR-138 in OSCCs

**Figure 11:**
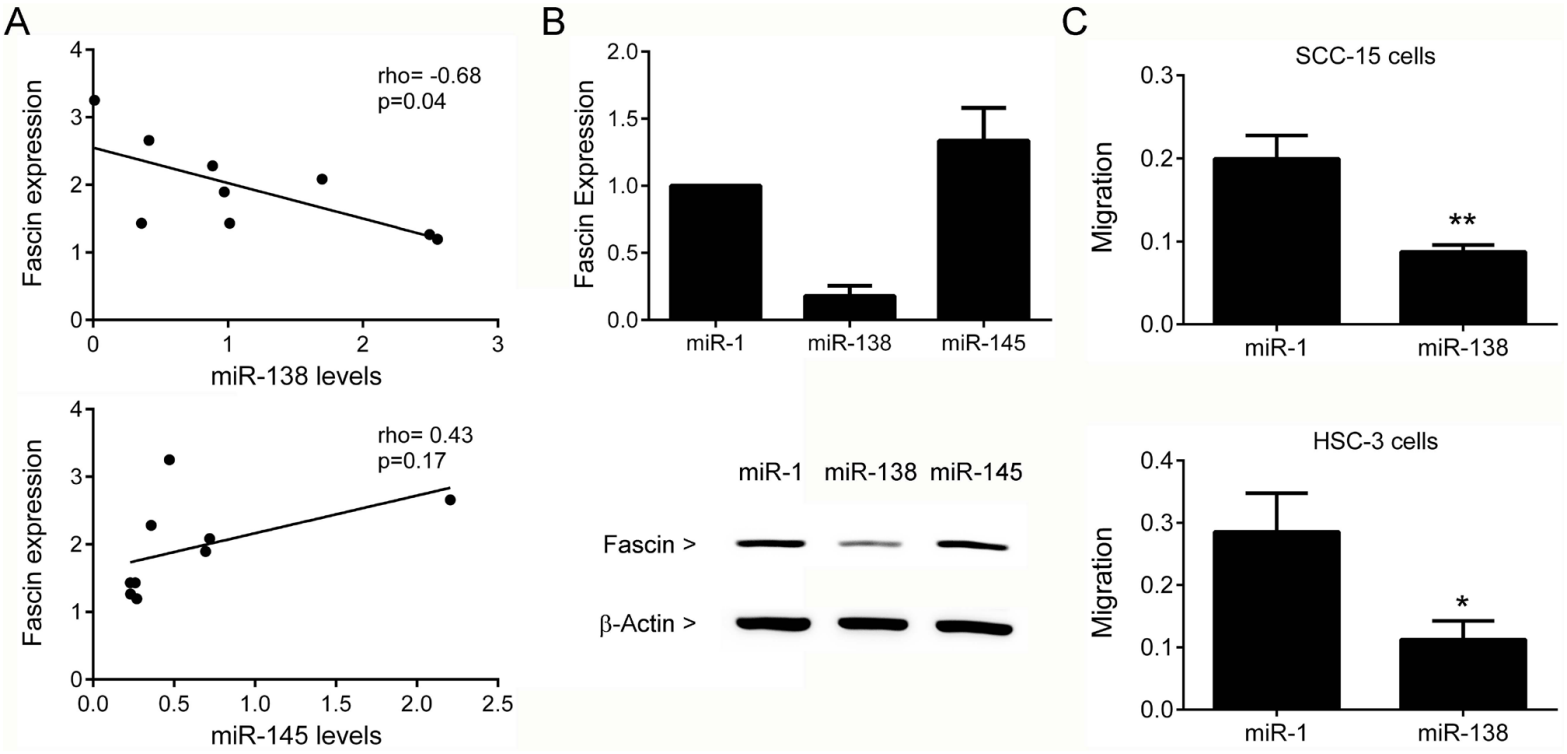
Fascin is target by miR-138 **(A)** Expression of fascin is inversely correlated with miR-138, but not with miR-145, in OSCC samples. **(B)** SCC-15 cells were exposed to miR-1 (scramble control) or miR-138 and miR-145 mimics. Levels of fascin mRNA and protein were clearly decreased in miR-138 transfectants, demonstrating that miR-138 regulates fascin mRNA levels. **(C)** Overexpression of miR-138 significantly inhibited the migration of SCC-15 (p<0.005) and HSC-3 (p<0.01) cells. *p<0.01, **p<0.005.

To gain insight into the molecular mechanism by which fascin is overexpressed in OSCCs, we determined the relationship between fascin mRNA levels and the expression of miR-138 and miR-145 in a series of OSCC fresh tumor samples. Previous studies showed that miR-138 is downregulated in OSCCs [19, 20] and in silico target analysis revealed a conserved miR-138-binding site within 3' UTR of fascin mRNA (www.targetscan.org). Similarly, previous studies revealed that miR-145 regulates fascin expression in several different tumors [21, 22]. miR-138 showed a significant and inverse correlation with fascin levels (rho=-0.68 and p=0.04), whereas no correlation was observed for miR-145 (Figure 11A). To determine whether miR-138 and miR-145 regulate fascin mRNA, HSC-3 cells were transfected with miR-138 and miR-145 mimics. Heterologous over-expression of miR-138, but not miR-145, resulted in a concomitant decrease in fascin mRNA and protein, suggesting that miR-138 has the capacity to regulate fascin expression in OSCC (Figure 11B). As expected, miR-145 overexpression clearly decreased activin A expression [23], confirming the efficiency of transfection (data not shown).

To explore the role of miR-138, we examined the effect of miR–138 in OSCC cell migration. As shown in Figure 11C, overexpression of miR-138 significantly decreased cell mobility compared with control in both SCC-15 (p=0.005) and HSC-3 (p=0.01). Therefore, these results indicated that miR-138 plays a crucial role in regulating the migration of OSCC cells via targeting fascin.

## DISCUSSION

Prognostic indicators are important to refine treatment and forecasting outcomes in patients with OSCC. The most predictive factors to define risk category in OSCCs are the clinical stage and histological grade [2–5]. None of the molecular markers assessed has so far been able to provide sufficiently accurate information to benefit patients [24]. Identification of protein expression profiles is important for understanding the mechanisms of oral tumorigenesis, as they could facilitate the development of new tools for the prevention, diagnosis, treatment and prognosis of OSCCs. We recently applied a proteomic-based approach to identify differentially expressed tumor proteins in microdissected OSCCs compared to normal oral mucosa, and identified 69 proteins upregulated in OSCC [18]. Herein we selected fascin and plectin, two of the most significant overexpressed proteins, for validation and further analysis. Both proteins are related to promotion of cytoskeletal dynamics, increased migratory and invasive capacity and thereby the potential for metastasis. The effects of fascin and plectin on OSCC biology are not completely defined.

In the present study we confirmed that fascin and plectin are significantly upregulated in OSCC compared with normal oral mucosa at gene expression and protein levels in patient samples. Fascin and plectin were also overexpressed in OSCC-derived cell lines. Subsequent immunohistochemical analysis of specimens representing OSCC development revealed that fascin levels were already significantly upregulated in mild dysplasias, keeping higher levels throughout oral cancer progression, whereas plectin upregulation was detected only in cancer areas. Further analysis showed that increased expression of fascin was significantly associated with shortened disease-specific survival for OSCC patients. Unexpectedly, plectin levels did not show statistical significance for survival in OSCC patients. In the multivariate Cox regression analyses, fascin emerged as an independent predictor factor of disease-specific survival along with consistent prognostic factors for OSCCs such as advanced age, tumors with high T classification and presence of cervical lymph metastasis (N stage) [25–27]. In the validation cohort (cohort 2), high levels of fascin were also significantly associated with worse prognosis, confirming fascin as a predictor of OSCC prognosis.

Earlier investigations also showed that fascin expression was increased in several types of cancer, including OSCC [12, 28], and the overexpression was an indicator of worse prognosis [12, 13]. Chen and collaborators [28] showed that high levels of fascin were associated with TNM clinical stage, but they did not show survival analysis. These authors mixed cancer located at oral and oropharyngeal sub-sites, which are proofed to be distinct regarding etiology and prognosis. The immunoexpression of fascin was significantly higher in dysplasia and *in situ* carcinoma than in benign diseases [29], reinforcing our findings that fascin can be helpful for improving the diagnostic accuracy of dysplasias that will progress to oral carcinoma. Two studies evaluated plectin as a prognostic marker in head and neck carcinomas, however, they presented results opposed to ours [17, 30]. Patients with higher levels of plectin showed a lower overall survival rate, which was probably related with the higher metastatic potential of the tumors with increased plectin level [17]. Nevertheless, these authors included tumors from different sites (oropharynx, hypopharynx, larynx and salivary glands), raising the question if the significance was not driven by tumor from sites different than oral cavity (site-dependency). Rikardsen and collaborators [30] found high expression of plectin correlated significantly with disease-specific-survival in all patients with non-metastatic disease.

Previous studies have shown that fascin, as an actin-binding and bundling protein, promotes cell migration and adhesion dynamics *in vitro* and tumor metastasis *in vivo*. Furthermore, fascin can directly interact with the microtubule cytoskeleton, which does not depend upon fascin-actin bundling, further contributing to regulation of focal adhesion dynamics and cell migration speed [31]. We generated OSCC cell lines stably expressing shRNA targeting fascin to understand the exact role of fascin in preclinical model. In agreement with other studies, fascin knockdown had no significant effects on viability and proliferative potential of OSCC cells [32, 33]. In contrast, overexpression of fascin increased proliferation of OSCC cells, which was accompanied by upregulation of PI3K and MAPK pathways [13]. The cell proliferation differences suggest that fascin may have distinct roles in the context of cell tumor origin. In accordance with previous studies [12–14], we also demonstrated that fascin plays a negative control on cell adhesion and regulate migration and invasion status of OSCC cells, which may explain why higher fascin expression was significantly correlated with worse prognosis. High fascin expression induced hepatocellular carcinoma cell invasion associated with loss of E-cadherin and gain of matrix metalloproteinases 2 and 9 levels [34]. EMT comprise the biological processes by which cells transit between epithelial and mesenchymal states and includes decreased cell-cell adhesion and increased cell motility and metastasis [35]. In cells depleted of fascin, we observed an induction of E-cadherin and a repression of vimentin, suggesting that overexpression of fascin promotes the EMT, an important phenotype for invasion and metastasis. The xenograft tumors in the flank were slighted smaller in the presence of fascin-silenced OSCC cells, but the orthotopic model showed no differences in the frequency of cervical lymph node metastasis between tumors formed by cells knockdown for fascin and their controls. This finding can be attributed to the high aggressive behavior of the OSCC cells (specifically HSC-3), since silencing the gene expression of fascin was not able to reduce the highly metastatic potential of this cell line. To provide a better understanding of the molecular events involved in the fascin-mediated OSCC progression is necessary additional *in vivo* experiments and mechanistic studies.

In the context of cell migration, the transport of adhesion proteins and receptors to filopodia tips has been implicated in the formation and reinforcement of cell-cell junctions [36, 37], contributing to cell locomotion [38]. Villari and collaborators [31] demonstrated that fascin may interact directly with the microtubule cytoskeleton independently of actin cooperation, to generate a complex with FAK and Src to control adhesion stability. Evidences demonstrated that filopodia shaft adhesions can mature into focal adhesions upon lamellipodia advancement [39], suggesting that focal adhesion proteins found at the shaft and/or tip of filopodia could be representative of nascent adhesions. Our results revealed reduced expression of paxillin and decreased phosphorylation of both paxillin and FAK following EGF stimuli in fascin-silenced cells. These findings support that fascin has a direct effect on filopodia organization in the context of focal adhesion formation during the invasion process.

Another interesting finding of the present study lies on the microRNA analyses. Our findings revealed that the expression of miR-138 is inversely correlated with fascin levels, and the transfection of miR-138 mimic in OSCC cells result in a downregulation of fascin, suggesting that fascin overexpression in OSCCs is, at least in part, regulated by this microRNA. On the other hand, no correlation between miR-145 and fascin transcripts was observed and ectopic expression of miR-145 did not alter significantly fascin at both mRNA and protein levels. miR-138 is frequently down-regulated in various tumors [19, 20, 40] and its reduced expression is associated with a shorter tumor-specific and relapse-free survival [41, 42]. It has been described that miR-138 has tumor suppressive functions, including regulation of proliferation, apoptosis, migration and invasion. In non-small-cell lung cancer, forced expression of miR-138 inhibited cell proliferation and reversed epithelial-mesenchymal transition via target of G-protein-coupled receptor kinase-interacting protein 1 and semaphorin 4C [43], and up-regulation of miR-138 inhibited hypoxia-induced cardiomyocyte apoptosis via down-regulating lipocalin-2 expression [44]. A previous study showed that miR-138 expression is involved in the multidrug resistance of leukemia cells through down-regulation of MDR1 [45]. However, a following study revealed that miR-138 modulates MDR1 expression indirectly by inhibiting NF-κB/p65, a well known transcriptional factor involved in MDR1 regulation [46]. Importantly, the multidrug resistance of human leukemia cell line HL-60 was achieved after overexpression of miR-138. Thus, loss of miR-138, which is an endogenous inhibitor of fascin, may promote aberrant expression of fascin and other targets, contributing to pathogenesis and progression of OSCCs.

In conclusion, fascin is upregulated in patients with OSCC and OSCC-derived cell lines, the overexpression of fascin is significantly correlated with disease progression and poor outcome, and fascin may have a major role in OSCC migration and invasiveness process. Further investigation is necessary to establish fascin as a routine therapeutic target for patients with OSCC.

## MATERIALS AND METHODS

### Tissue samples

The study was approved by the Human Research Ethics Committee of the School of Dentistry, University of Campinas (protocol number: 090/2011). This study included different sets of human samples. Initially, to confirm the higher expression of fascin and plectin in OSCC samples compared with healthy oral mucosa, we performed immunohistochemical analysis in the ten pairs of samples used in the original LC-MS/MS screening [18]. To expand the number of samples and characterize further the expression pattern of fascin and plectin, fresh samples of OSCC (n=11) and normal oral mucosa (n=11) were used to investigate the expression of fascin and plectin using real time quantitative PCR (qPCR) [47]. Those samples were also used for the expression of miR-138 and miR-145, putative microRNA regulators of fascin mRNA.

Immunohistochemical analysis was also performed in two independent sets of samples characterizing OSCC development. Cohort 1 was composed by 29 cases of oral fibrous hyperplasias with normal epithelium, 24 mild epithelial dysplasia, 26 moderate dysplasia, 19 severe dysplasia and 113 primary OSCCs. Oral epithelial dysplasias and OSCCs were classified according to the World Health Organization (WHO) grading system. Clinicopathological features of these 113 OSCC patients who were diagnosed and treated from 1998 to 2008 at Oncology Center of Cascavel (CEONC, n=46 patients) and UOPECCAN Cancer Hospital (n=67 patients) were described [5]. Cohort 2 was composed by 52 primary OSCCs diagnosed and treated at Jewish General Hospital, McGill University [48]. The samples were contained in a tissue microarray (TMA) and were used to validate the fascin findings.

### Cell cultures

Normal human gingival keratinocyte cell line (HGK) was cultured in serum-free, low calcium media (Gibco’s Keratinocyte-SFM; Invitrogen, USA) containing specific supplements and antibiotics [49]. The human OSCC cell lines SCC-4, SCC-9, SCC-15 and SCC-25 were obtained from American Type Culture Collection (ATCC, Manassas, VA, USA), and cultured as recommended in a 1:1 mixture of Dulbecco’s modified Eagle’s media and Ham’s F12 media (DMEM/F12; Invitrogen, USA) supplemented with 10% fetal bovine serum (FBS), 400 ng/ml hydrocortisone (Sigma-Aldrich, USA) and antibiotics. The SCC-9 ZsGreen LN-1 cell line, isolated from a metastatic cervical lymph node, was previously described [50], and cultured in the same conditions as the parental SCC-9 cell line. HSC-3, a human tongue squamous cell carcinoma cell line (JCRB 0623; Osaka National Institute of Health Sciences, Japan), was cultured in DMEM/F-12 media (Invitrogen, USA) supplemented with 10% FBS, 50 μg/ml ascorbic acid (Sigma-Aldrich, USA), 400 ng/ml hydrocortisone (Sigma-Aldrich, USA) and antibiotics. All cells were growth at 37°C in a humidified atmosphere of 5% CO_2_.

### Immunohistochemistry

Immunostaining of fascin and plectin was performed using the streptavidin-biotin peroxidase complex method. Briefly, after dewaxing and hydration in graded alcohol solutions, the sections were treated with 3% H_2_O_2_ followed by antigen retrieval with 10 mM citric acid pH 6.0 in a pressure cooker. After washing with phosphate-buffered saline (PBS), the sections were treated with 1% bovine serum albumin (BSA) in PBS for 1 h and then incubated with monoclonal mouse antibody against fascin (clone IM20; Abcam Inc, USA), diluted 1:700, or polyclonal goat antibody against plectin (clone C-20; Santa Cruz Biotechnology Inc, USA), diluted 1:200, followed by the LSAB method (LSAB+ System-HRP kit, Dako, USA). Reactions were developed by incubating the sections with 0.6 mg/ml 3,3'-diaminobenzidine tetrahydrochloride (Sigma-Aldrich, USA) containing 0.01% H_2_O_2_. Control reactions were performed by omission of the primary antibody (Supplementary Figure 6). Immunoexpression was assessed with the aid of the Aperio ScanScope CS (Aperio Technologies, USA), as previously described [23].

The TMA immunostaining (OSCC cohort 2) was performed in the Nexus Immunostainer (Ventana Medical Systems, USA) using antibodies against fascin (1:100, clone 55K2; Santa Cruz Biotechnology, USA). Each core was scanned and protein expression was assessed using a four-tiered system as previously described [48]. For statistical analysis, samples were categorized into two groups: negative/weak (low expression) and moderate/strong (high expression).

### qPCR

Total RNA from fresh tissues and cell lines was isolated with the RNeasy mini kit (Qiagen, USA) according to the manufacturer's protocols. Following DNase I treatment in order to eliminate genomic DNA contamination, 1 μg of total RNA per sample was used to generate cDNA using Oligo-dT (Invitrogen, USA) and reverse transcriptase (Superscript II RT enzyme, Invitrogen, USA). The resulting cDNAs were subjected to qPCR using specific primers and SYBR^®^ Green PCR master mix (Applied Biosystems, USA) in the StepOnePlus Real Time PCR (Applied Biosystems, USA). Gene expression was determined using the 2^-ΔΔCt^ method and the housekeeping gene *PPIA* (cyclophilin A) was used as reference gene for data normalization. All reactions were performed in triplicate. The primers used in this study are as follows: *FSCN1* (fascin) forward 5’GGCGAGTCTGGCACCTCTT3’ and reverse 5’CCCCAACCGTCCCTTAGC3’, *PLEC* (plectin) forward 5’GGAGGATGCGTTTCCACAA3’ and reverse 5’ATATCTGAGATCTGGAAGTGCAGAA3’, and PPIA forward 5’GCTTTGGGTCCAGGAATGG3’ and reverse 5’GTTGTCCACAGTCAGCAATGGT3’.

### Stable cells mediating fascin silence

Lentiviral vectors containing short hairpin RNA (shRNA) targeting human fascin (FSCN1 MISSION^®^ shRNA Lentiviral Transduction Particles-SHCLNV-NM_003088) or scrambled control shRNA (MISSION^®^ pLKO.1-puro Non-Mammalian shRNA Control) were prepared by Sigma-Aldrich (USA). SCC-15 and HSC-3 cells grown in a 12-well plate at confluence of 70% were incubated with control or fascin shRNA lentiviral particles at a multiplicity of infection (MOI) of 1.5 in culture media containing 8 mg/ml of polybrene (Sigma-Aldrich, USA) for 4 h. After washing with PBS, cells were cultured in fresh media for an additional period of 48 h. Cells were then cultured for 10 days in the presence of 1 μg/ml of puromycin dihydrochloride (Sigma-Aldrich, USA) to select resistant cells. The efficacy of fascin knockdown was determined by qPCR and western blot.

### Western blot analysis

Cells were washed with cold PBS and lysed in a protein-lysis buffer containing 10% sucrose, 1% NP-40, 20 mM Tris-HCl (pH 8.0), 137 mM NaCl, 10% glycerol, 2 mM EDTA and a cocktail of protease inhibitors (Roche Diagnosis, USA). After centrifugation, protein concentrations were measured using a protein assay according to the manufacturer’s instructions (Bio-Rad Protein Assay, Bio-Rad, USA). Thirty μg of total protein per sample were resolved in a 10% sodium dodecyl sulphate polyacrylamide gel electrophoresis (SDS-PAGE) under reducing conditions, and transferred to nitrocellulose membranes. The membranes were blocked with 10% non-fat dry milk in PBS containing 0.1% Tween-20, rinsed in the same buffer, and incubated for 2 h with monoclonal mouse antibody against fascin at 1:1,000 (clone IM20; Abcam Inc, USA) or monoclonal mouse antibody against β-actin (clone AC-15; Sigma-Aldrich, USA) at 1:50,000. After washing, the membranes were incubated with anti-mouse IgG fluorescein-conjugated (Cell Signaling, USA), and signals captured with an Alliance 9.7 instrument (UVITEC, Cambridge, UK).

### Cell viability and cell proliferation assays

Cell viability and cell proliferation assays were performed according to Sobral [51]. Three independent experiments were performed with five replicates.

### Adhesion assay

Adhesion analysis was performed as described previously [52], using fibronectin (BD Biosciences, USA) or myogel [53] as substrate.

### Migration and invasion assays

The migratory potential of the fascin silenced-cells was assessed by both transwell migration assay and scratch wound migration assay (IncuCyte™ live-cell imaging system - Essen BioSciences), whereas invasion assays were based in the transwell with myogel solidified with low-melting agarose [53] and in the human myoma organotypic culture [54].

### Analysis of EMT markers E-cadherin and vimentin

The expression of the epithelial marker E-cadherin and of mesenchymal marker vimentin was carried out using qPCR as previously described [47].

### Quantification of filopodia

HSC-3 shRNA FSCN and HSC-3 shRNA control cells were fixed in 1% paraformaldehyde in PBS containing 0.25% Triton X-100 for 15 min, and then were incubated with rhodamine phalloidin (diluted 1:100; Invitrogen, USA) for 1 h. Quantification of filopodia was performed with images captured with a fluorescent microscope (Axiophot; Carl Zeiss MicroImaging Inc, Germany).

### Analysis of filopodia-related proteins

Paxillin, focal adhesion kinase (FAK) and vinculin expression in fascin-silenced HSC-3 cells and controls was assessed by western blot, using the following antibodies: anti-paxillin (diluted 1:500; Abcam Inc, USA), anti-phospho paxillin (diluted 1:500; Cell Signaling, USA), anti-FAK (diluted 1:500; Millipore, USA), anti-phospho-FAK (diluted 1:500; Cell Signalling, USA), anti-vinculin (diluted 1:500; Sigma-Aldrich, USA), anti-GAPDH (diluted 1:500; Sigma-Aldrich, USA). Western blot with phospho-fascin (1:50, clone FP2661; ECM Bioscience, USA) was also performed.

To assess the effects of epidermal growth factor (EGF) stimuli on paxillin and FAK phosphorylation, HSC-3 shRNA Control and HCS-3 shRNA FSCN cells were starved and cultured for 5, 15 and 30 min with media containing 50 ng/ml of EGF (Sigma-Aldrich, USA).

### Confocal microscopy

HSC-3 cells grown on glass coverslips for 24 h were fixed in 1% paraformaldehyde in PBS containing 0.25% Triton X-100 for 15 min. The cells were then washed twice with PBS and blocked with blocking buffer (1% BSA and 2% normal goat serum in PBS) for 30 min. Antibodies targeting fascin (clone 55K2; Santa Cruz Biotechnology, USA), diluted 1:500, and paxillin (Abcam Inc, USA), diluted 1:100, were added. The cells were washed 3 times in PBS and incubated in blocking buffer containing respective Alexa-conjugated secondary antibodies (Invitrogen, USA) at 1:1000 and incubated for 1 h at room temperature. Cells were mounted with Vectashield containing DAPI (Vector Labs, USA). Images were captured using a 40x or 60x oil immersion objectives on the Wave FX spinning disk confocal microscopy system (Quorum Technologies) and analyzed using Volocity.

### Tumorigenicity assay

To assess the growth of xenograft tumors in nude mice, HSC-3 shRNA Control and HSC-3 shRNA FSCN were injected subcutaneously in the flank or were implanted into the tongue (orthotopic model) of 12 week-old BALB/c nude mice (20 animals/cell line). Animals were sacrificed 3 weeks later and the tumors in the dorsum were dissected, weighted and fixed in 10% formalin for hematoxylin and eosin (HE) stain and immunohistochemistry. Animals, which received the injection of the tumor cells in the tongue, had the tongue and cervical lymph nodes dissected, fixed and subjected to HE stain and immunohistochemistry. Primary tumors (both in flank and tongue) were subjected to immunohistochemical analysis using the anti-fascin antibody, whereas cervical lymph nodes were analyzed with anti-pancytokeratin (clone AE1/AE3; Dako, USA).

### Correlation between fascin and miR-138 and miR-145 expression

The expression of miR-138 and miR-145 was assessed in fresh tumor specimens. Briefly, 1 μg of total RNA was converted into specific cDNA derived from mature microRNAs using TaqMan microRNA Reverse Transcription Kit (Applied Biosystems, USA) and quantified in triplicate using the TaqMan microRNA assay. The small nucleolar RNA (snoRNA) RNU48 was used as endogenous control. All assays were obtained from Applied Biosystems through their Assay-on-Demand service. Data were quantified and analyzed using sequence detection system (version 2.3) (Applied Biosystems, USA). The microRNA relative expression in fresh tumor specimens was normalized against endogenous control and pooled normal oral mucosa samples.

### Effect of miR-138 and miR-145 mimics on fascin expression

HSC-3 cells were transfected with miR-138 or miR-145 mimics using the RNAiMAX reagent (Invitrogen, USA) as per the manufacturer's instructions. As control, cells were transfected with an unspecific scramble sequence (Pre-miR Negative Control #1-miR-1, Life Technologies, USA). After 72 h, cells were harvested and subjected to qPCR and western blot for quantification of fascin as described above.

### Statistical analysis

Differences on expression of fascin and plectin between OSCC and normal oral mucosa samples were analyzed using the Mann-Whitney U test. To assess the immunohistochemical expression of fascin and plectin in the set of samples characterizing OSCC progression, Kruskal-Wallis test was applied. Correlations between immunohistochemical expression of fascin and plectin and clinicopathological parameters of the tumors were performed using Spearman’s rank correlation. Survival curves were constructed based on the Kaplan-Meier method and compared with the Log-rank test. For multivariate survival analysis, the Cox proportional hazard model with a stepwise method including all parameters was employed.

All *in vitro* assays were performed at least three times. Mann-Whitney U test or one-way analysis of variance (ANOVA) with post-hoc comparisons based on the Tukey's multiple comparisons test were applied. The level of significance considered was 5% (p≤0.05).

## SUPPLEMENTARY MATERIALS FIGURES


