# Supplementary material for: Fascin promotes migration and invasion and is a prognostic marker for oral squamous cell carcinoma
Source: Oncotarget. 2017 Aug 19;8(43):74736–54. doi: 10.18632/oncotarget.20360 (PMC5650375; doi:10.18632/oncotarget.20360)
Supplement: Supplementary file 1 [file oncotarget-08-74736-s001.pdf]

Fascin promotes migration and invasion and is a prognostic marker for oral squamous cell carcinoma

SUPPLEMENTARY MATERIALS

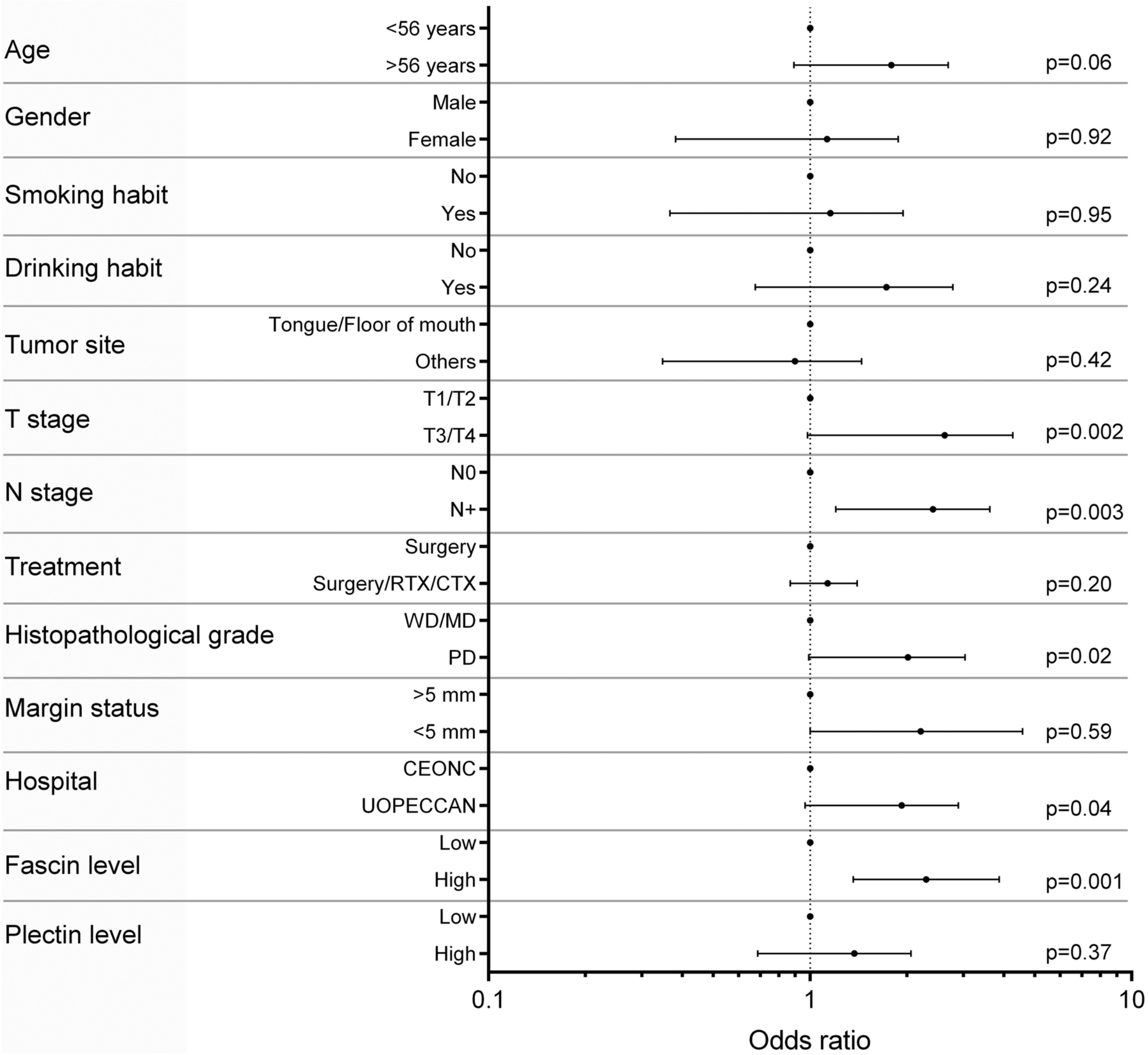

Supplementary Figure 1: Disease-specific survival rates of the OSCC patients according to clinicopathological and immunohistochemical parameters.

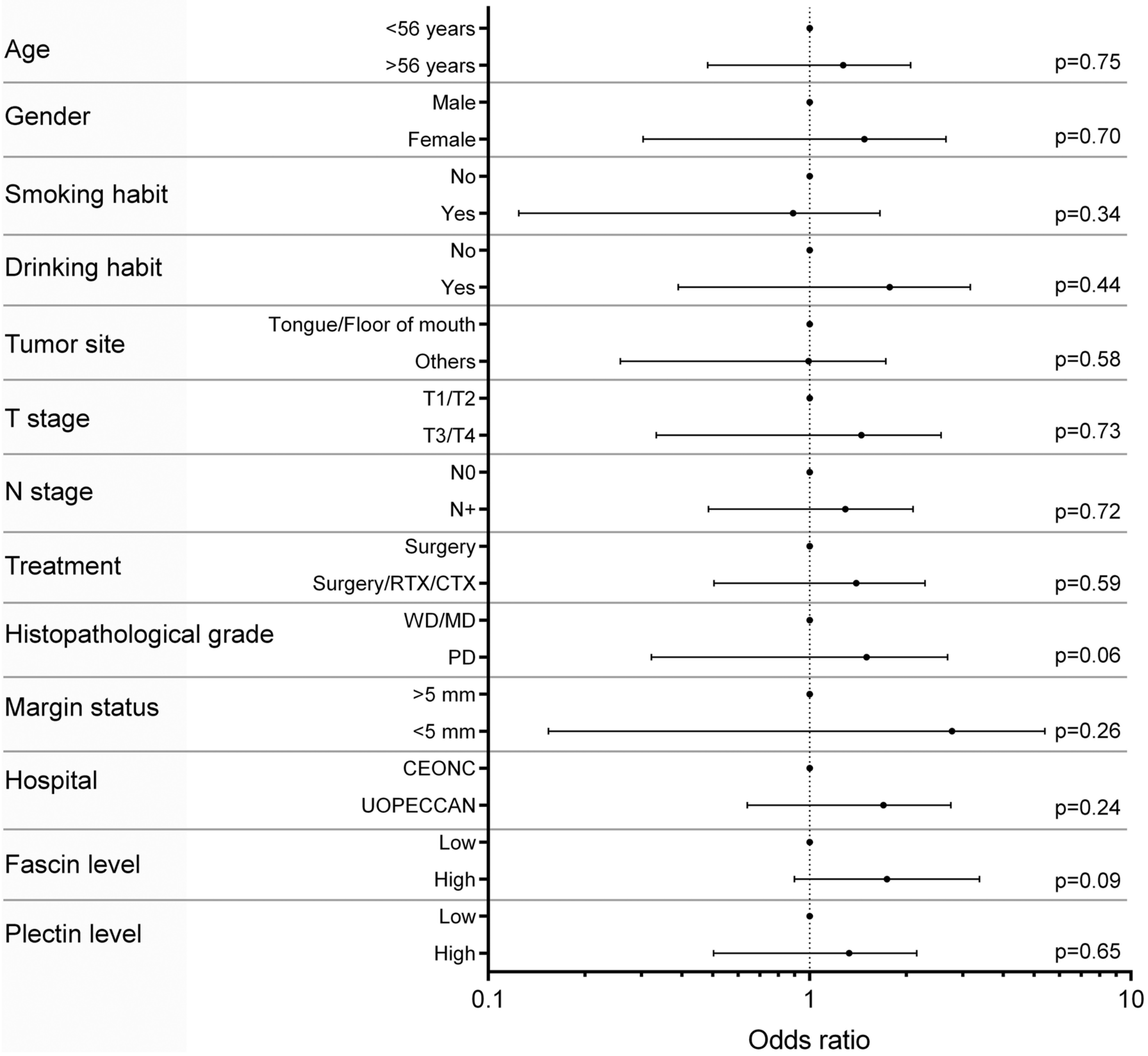

Supplementary Figure 2: Disease-free survival rates of the OSCC patients according to clinicopathological and immunohistochemical parameters.

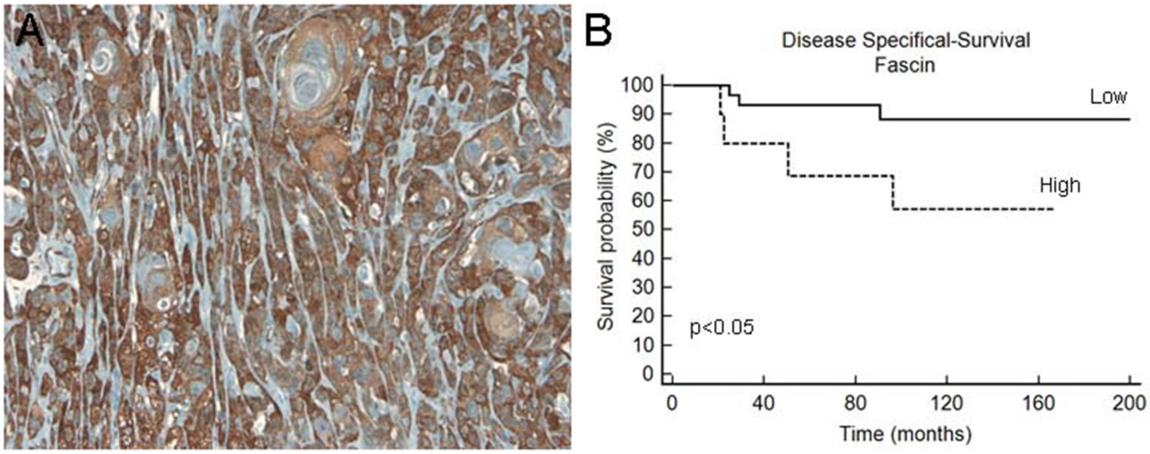

Supplementary Figure 3: Immunohistochemistry analysis of fascin in OSCC cohort 2. **(A)** Representative image of immunostaining for fascin using the anti-fascin antibody clone 55K2 (Santa Cruz Biotechnology, USA). A clear cytoplasmic staining for fascin was observed. **(B)** The survival curve, analyzed by Kaplan-Meier test, revealed a short survival rate in samples with high levels of fascin.

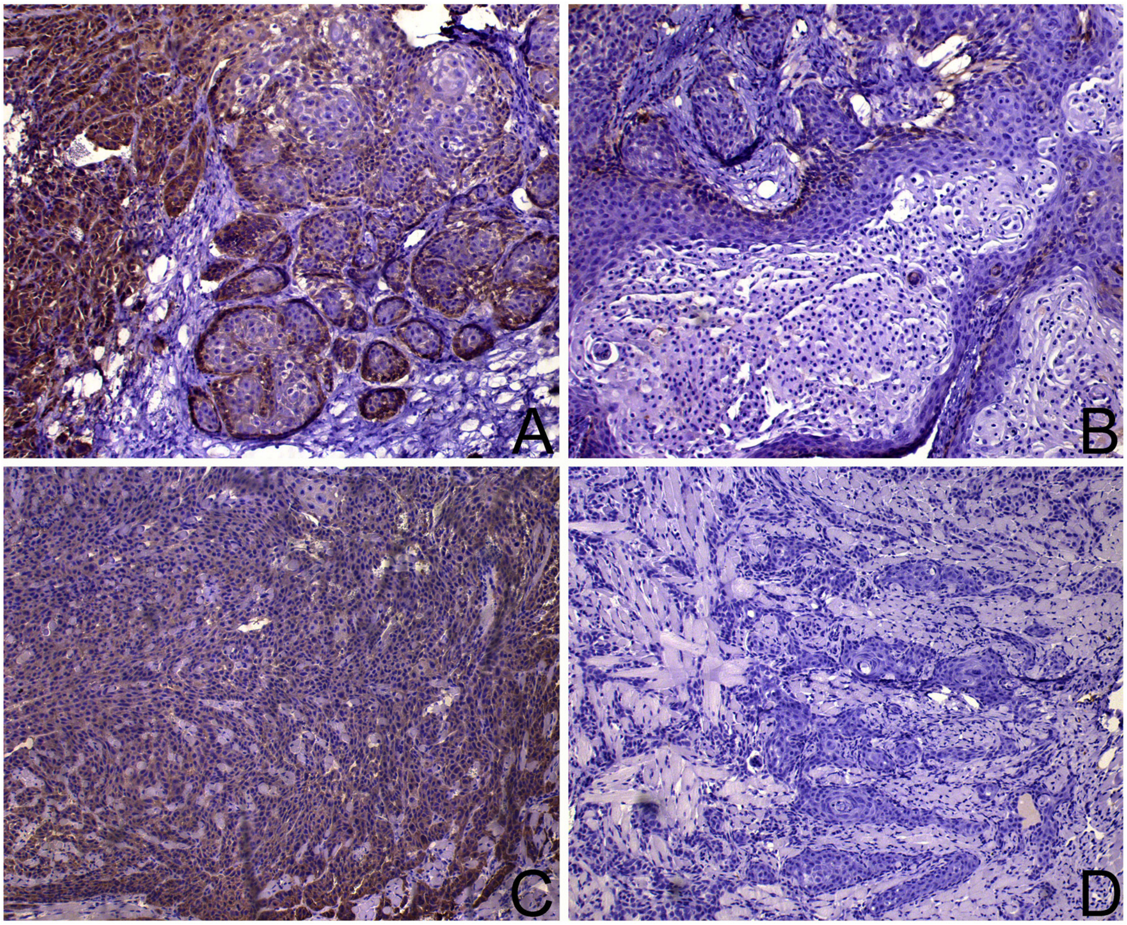

Supplementary Figure 4: Representative immunohistochemical images of the xenograft tumors formed by HSC-3 shRNA Control cells (**A** and **C**) and HSC-3 shRNA FSCN cells (**B** and **D**). Fascin expression was reduced in the HSC-3 shRNA FSCN tumors compared with the HSC-3 shRNA Control tumors.

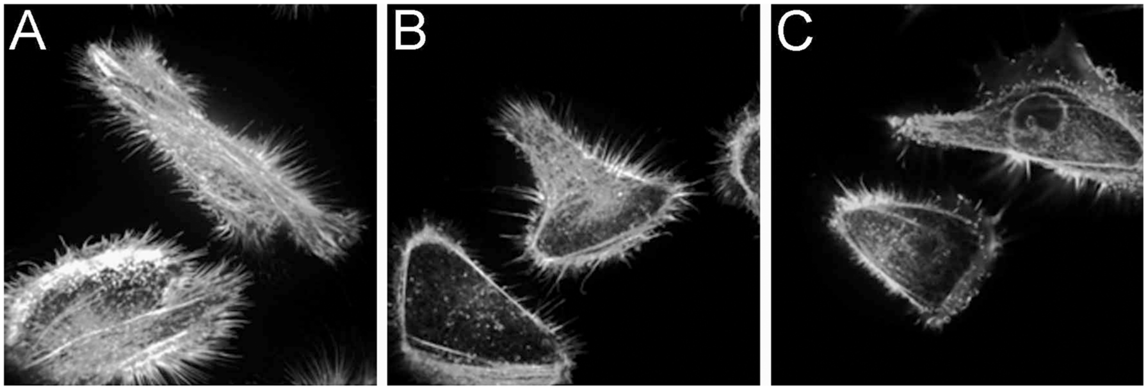

Supplementary Figure 5: Detection of filopodia in HSC-3 **(A)**, HSC-3 shRNA Control **(B)** and HSC-3 shRNA FSCN **(C)**. Cells were labeled with rhodamine phalloidin to characterization of actin filaments. Filopodia were more abundant in HSC-3, HSC-3 shRNA Control than in HSC-3 shRNA FSCN.

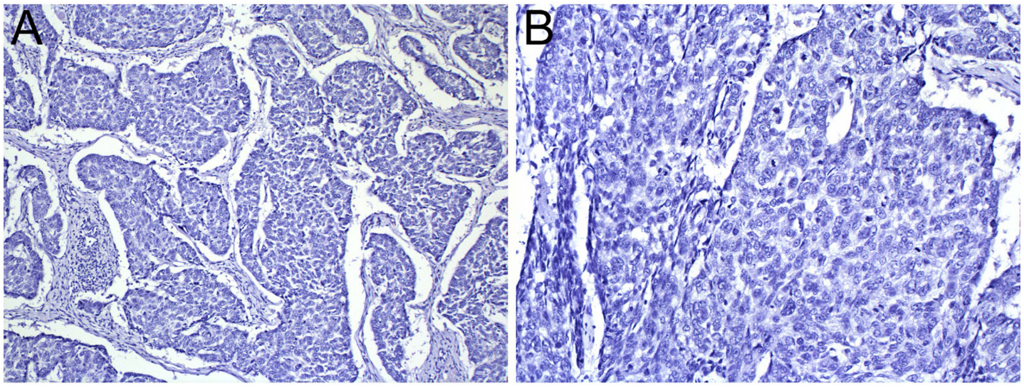

Supplementary Figure 6: Negative control of the immunohistochemistry reaction. The negative control represents the tissue (oral squamous cell carcinoma sample) incubated with antibody diluent, without one of the primary antibodies. **(A)** original magnification of 100x and **(B)** high power view of (A) (original magnification of 200x).
